# Oxygen Gas Sensing with Photothermal Spectroscopy in a Hollow-Core Negative Curvature Fiber

**DOI:** 10.3390/s20216084

**Published:** 2020-10-26

**Authors:** Yingzhen Hong, Haihong Bao, Wei Jin, Shoulin Jiang, Hoi Lut Ho, Shoufei Gao, Yingying Wang

**Affiliations:** 1 Department of Electrical Engineering, The Hong Kong Polytechnic University, Hung Hom, Kowloon 999077, Hong Kong, China; yingzhen.hong@connect.polyu.hk (Y.H.); edward.bao@connect.polyu.hk (H.B.); eehlho@polyu.edu.hk (H.L.H.); jngaofei@gmail.com (S.G.); 2 Photonics Research Center, The Hong Kong Polytechnic University Shenzhen Research Institute, Shenzhen 518057, China; shoulin.jiang@polyu.edu.hk; 3 Institute of Photonics Technology, Jinan University, Guangzhou 510632, China; wangyy@jnu.edu.cn

**Keywords:** optical fiber sensor, oxygen detection, photothermal interferometry, hollow-core fiber

## Abstract

We demonstrate a compact all-fiber oxygen sensor using photothermal interferometry with a short length (4.3 cm) of hollow-core negative curvature fibers. The hollow-core fiber has double transmission windows covering both visible and near-infrared wavelength regions. Absorption of a pump laser beam at 760 nm produces photothermal phase modulation and a probe Fabry-Perot interferometer operating at 1550 nm is used to detect the phase modulation. With wavelength modulation and first harmonic detection, a limit of detection down to 54 parts per million (ppm) with a 600-s averaging time is achieved, corresponding to a normalized equivalent absorption of 7.7 × 10^−8^ cm^−1^. The oxygen sensor has great potential for in situ detection applications.

## 1. Introduction

Sensitive detection of oxygen gas is important for a range of applications, such as identifying potential air-to-fuel ratio variations in combustion systems [1] and monitoring the performance of aircraft fuel tank systems in the aerospace industry [2]. Conventional electrochemical oxygen sensors suffer from aging and require the use of reference gas and/or periodic calibration, while paramagnetic sensors have low sensitivity and low stability in vibration environments [3]. There are considerable research interests in optical oxygen sensors based on fluorescence quenching using fluorescence materials in combination with optical fibers. However, fluorescent materials are expensive, and efficient collection of fluorescence still needs to be addressed [4].

Laser absorption spectroscopy (LAS), as a highly selective and sensitive spectroscopic technique, has been studied for oxygen sensing. Oxygen has absorption lines around 760 nm, which have little overlap with the absorption bands of other gases. In 2014, Neethu et al. demonstrated oxygen detection using wavelength modulated tunable diode laser absorption spectroscopy [5]. By using a 56-cm-long gas cell and detecting the ratio of the second to the first harmonic signal, they achieved a limit of detection (LOD) of 6500 parts per million (ppm). In 2017, Zhou et al. employed a 35-cm cubic diffuse integrating cavity to increase the absorption path length and achieved an oxygen sensitivity of 350 ppm and an uncertainty of 0.05% [6]. In 2019, Jatana et al. demonstrated oxygen detection in high-temperature gas streams utilizing an 8.5-cm-long Herriott cell with ~4.4 m effective absorption path length and achieved LOD of 1000 ppm [1].

The development of low-loss hollow-core fibers (HCFs) enables strong light-gas interaction in the hollow-core over a long distance, providing the possibility of remote and distributed gas sensing. In 2015, Munzke et al. demonstrated an oxygen sensor using a 10-m-long hollow-core photonic bandgap fiber (HC-PBF) placed between two high reflectivity mirrors to form an optical resonator with an effective path length of 70 m [7]. By measuring the ring-down time of the resonator, an estimated LOD of 11,000 ppm was achieved.

Photothermal interferometry (PTI) is a derivative of LAS and is a highly sensitive spectroscopic technique for trace gas detection [8]. The photothermal (PT) process involves localized heating generated from the relaxation of the ro-vibrational state of gas molecules via molecular collisions [9]. Pump absorption of gas molecules induces heating and modulates the refractive index of gas materials, which can be detected by measuring the phase modulation of a probe beam propagating through the material. Compared with conventional LAS, the PT phase modulation is proportional to pump power, which may be significantly enhanced by using a higher pump power. Phase detection with optical interferometry is more complex than intensity detection but provides higher sensitivity and larger dynamic range. In addition, PTI is background-free, with pump modulation contributing negligibly to the non-absorption background of the detected signal, which reduces the system noise.

Recently, PTI has been implemented with HCFs [10,11,12]. HCFs enable compact gas cells with a long optical path, and hence, high detection sensitivity. With a telecom band HC-PBF, detection of acetylene down to parts per billion (ppb) level has been demonstrated [10]. Compared to HC-PBFs, hollow-core negative curvature fibers (HC-NCFs) have simpler structures with an inverted curvature in the core wall [13], and much broader low-loss transmission windows [14]. With a HC-NCF capable of transmitting both near and mid infrared signals, detection of carbon monoxide down to ppm level has been demonstrated [15].

In this paper, we report a compact PTI-based oxygen sensor using a HC-NCF with double transmission windows covering both visible and near-infrared wavelengths. With a 4.3-cm-long HC-NCF in a low-finesse Fabry-Perot configuration as the gas cell, we demonstrated an all-fiber oxygen detection down to 54 ppm, corresponding to normalized equivalent absorption (NEA) of 7.7 × 10^−8^ cm^−1^.

## 2. Principle

### 2.1. Design and Fabrication of Gas Cell

**Figure 1:**
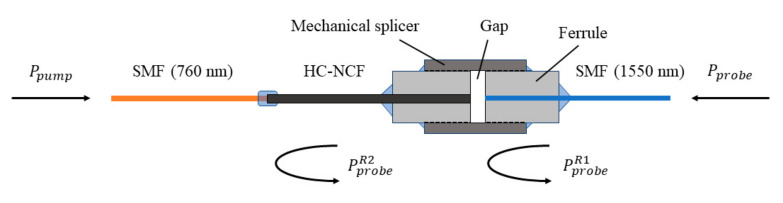
Schematic of the SMF/HC-NCF/SMF sensing unit. $P_{probe}^{R1}$ and $P_{probe}^{R2}$ are the reflected probe beams at the SMF/HC-NCF joints.

The sensing unit or gas cell is made of a piece of HC-NCF, as shown in Figure 1. One end of the HC-NCF is connected to a single mode fiber (SMF) at the pump wavelength (~760 nm) by fusion splicing, and the other end is butt-coupled to a SMF at the probe wavelength (~1550 nm) via a ceramic sleeve and ferrules, which are fixed together with ultraviolet curing glue. There is an air gap (<1 µm) at the HC-NCF/SMF butt-coupled joint to facilitate gas filling to the HC-NCF.

The modulated pump beam is delivered to the HC-NCF via the pump SMF (760 nm) to produce PT phase modulation. The amplitude of phase modulation ΔΦ may be expressed as [10]:

(1) $$\Delta \Phi =k\alpha \left(\lambda_{pump}\right)CLP_{pump},$$

where *k* is a coefficient which is fiber-specific; $\alpha \left(\lambda_{pump}\right)$ is the absorption coefficient for 100% oxygen concentration; *C* is the relative oxygen concentration; *L* is the length of the HC-NCF; $P_{pump}$ is the pump power delivered into the HC-NCF.

The probe beam is coupled into the HC-NCF from the opposite side via the probe SMF (1550 nm), and the reflections (~4%) at the two HC-NCF/SMF joints form a low-finesse Fabry-Perot interferometer (FPI) to detect the PT phase modulation. The phase detected is actually the phase difference between the reflected probe waves from the two fiber joints, which is twice the phase modulation given by Equation (1). Such an arrangement allows the complete separation of the pump and probe transmission optics while sharing the same HC-NCF gas cell, which allows the use of the best quality fibers in terms of transmission loss and mode quality and other components optimized for the pump and probe wavelengths, respectively. The fabricated FPI with SMF pigtails is packaged in a 3D-printed compact gas cell with two ports for gas in and out.

**Figure 2:**
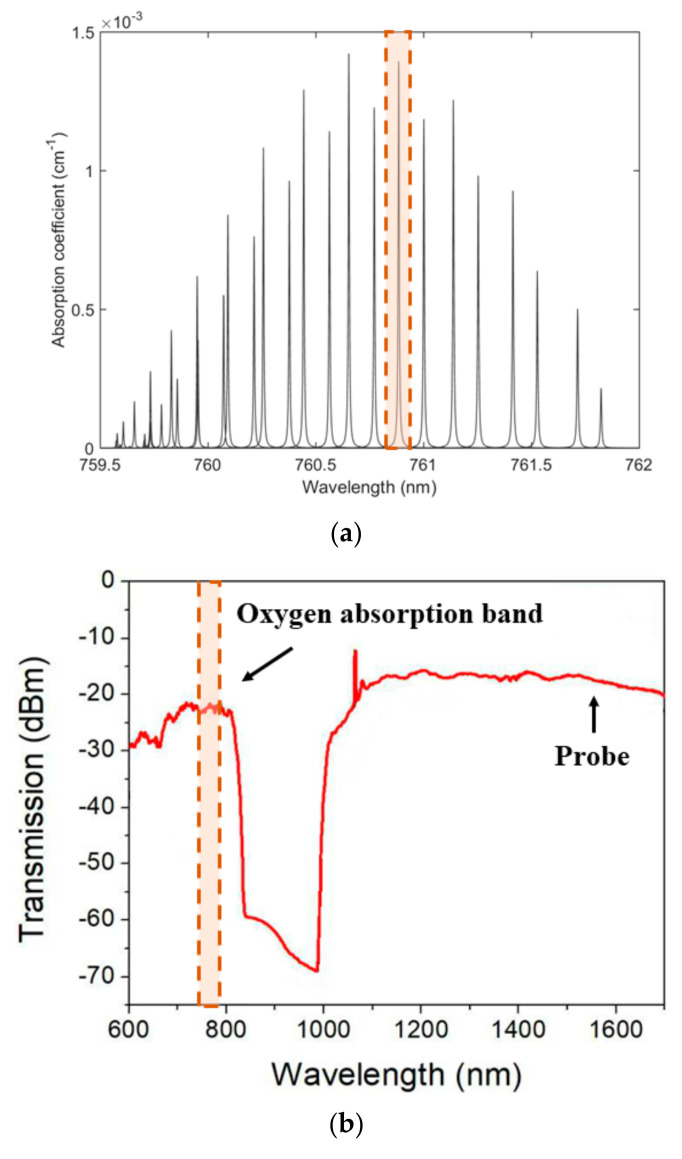

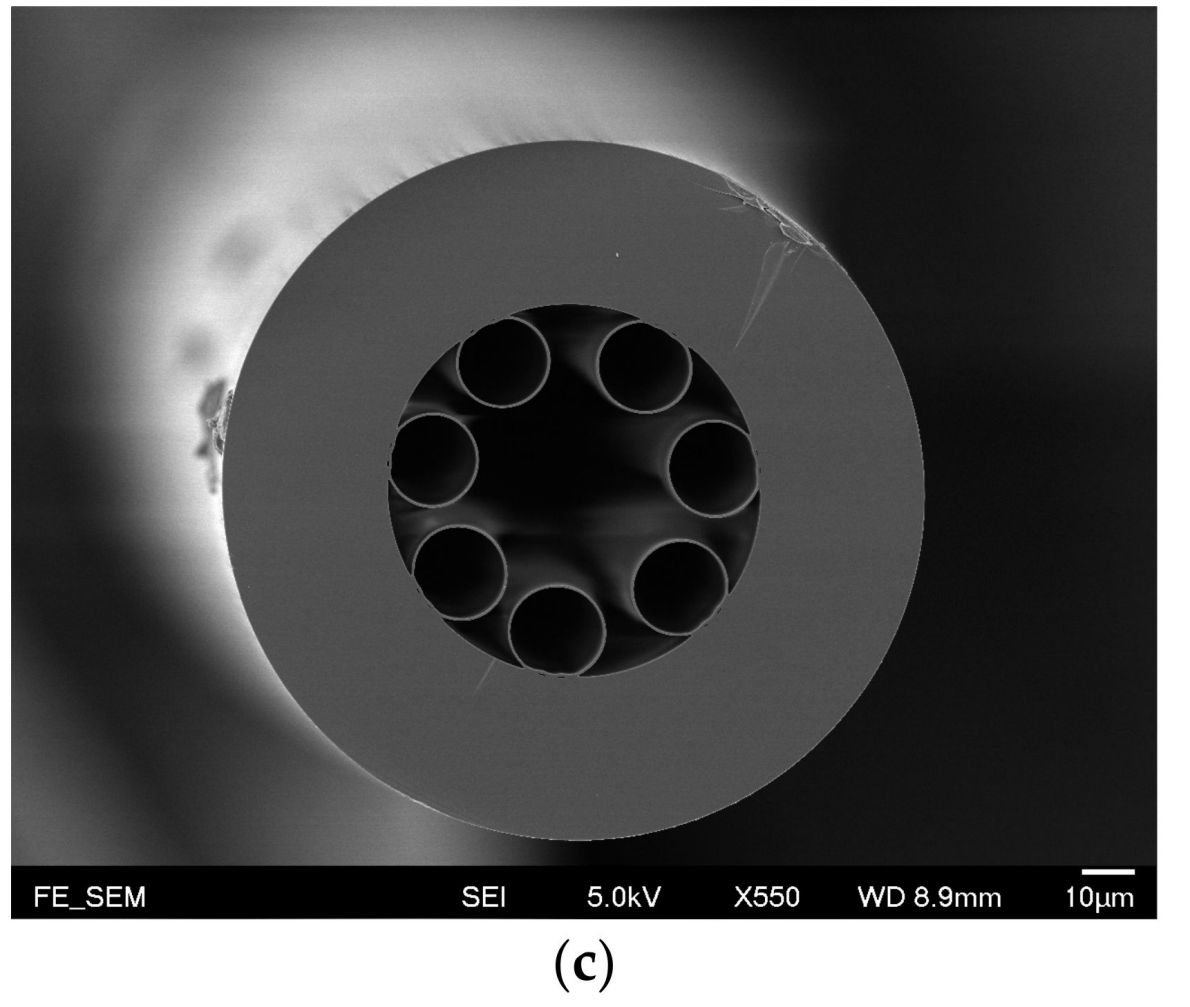
(**a**) Absorption lines of oxygen from 759.5 to 762 nm at 293 K and 1 atm determined by the HITRAN database. (**b**) Spectral transmission of the HC-NCF. (**c**) The cross-sectional image of the HC-NCF.

The absorption spectrum of oxygen around 760 nm is shown in Figure 2a. We used the absorption line around 760.88 nm, which had an absorption coefficient of 1.425 × 10^−3^ cm^−1^ at 293 K and 1 atm for a relative concentration of 100% [16]. The transmission spectrum of the HC-NCF used in this work is shown in Figure 2b. As shown in Figure 2c, the HC-NCF has an inscribed air-core with diameter of ~35 μm, which is surrounded by seven capillary rings with diameter of 17.5 μm. The HC-NCF has double transmission windows covering wavelength from below 600 to ~800 nm and from ~1 to beyond 1.7 µm. We operate the pump laser at 760.88 nm to produce PT phase modulation and use a probe laser at 1550 nm to detect the phase modulation.

### 2.2. PT Phase Modulation in the HC-NCF

The magnitude of PT phase modulation in the HC-NCF is dependent on the structure of the HC-NCF, pump modulation frequency, gas thermal relaxation rate, and thermal conduction parameters [17]. The relaxation in the PT process involves the multi-step transitions at a different relaxation rate. If the thermal relaxation rate is slower compared to the thermal conduction, the heat production may not be observed via PT signal [9]. For oxygen molecules, we focused on one characteristic time of the order of microseconds corresponding to the transition from $b^{1}\sum \begin{array}{c} +\\ g \end{array}$ to $a^{1}\Delta_{g}$, since the relaxation times of other processes are relatively long and above the order of milliseconds. In this case, only a part of the absorbed energy eventually contributes to the PT signal [18]. The efficiency of heating may be expressed in the form of [19]:

(2) $$H\left(\omega \tau \right)=\frac{1}{\sqrt{1+{\left(\omega \tau \right)}^{2}}},$$

where ω is the angular modulation frequency of pump beam and τ is the relaxation time. If the relaxation time is shorter compared to the pump modulation period, the absorbed energy will be effectively transferred to heat. However, when the value of ωτ is larger than or comparable to the unity, the efficiency of heating will be significantly reduced.

**Figure 3:**
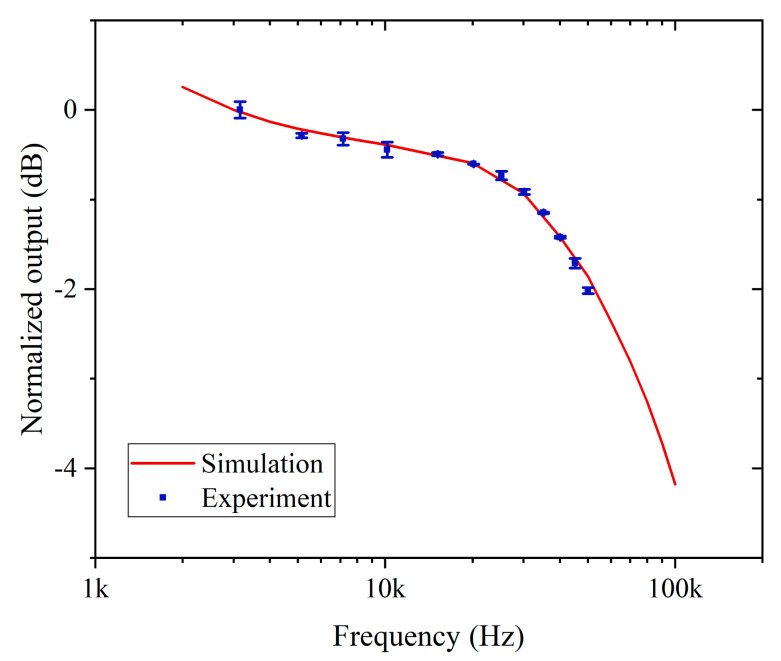
Normalized PT signal as a function of pump modulation frequency. The red line is calculated by COMSOL Multiphysics and the blue dots are the experimental data. Error bars show the standard deviation (s.d.) of measured data.

On the basis of considering the relaxation time and heating efficiency of oxygen, we further investigate the PT phase modulation in the HC-NCF. By using COMSOL Multiphysics software, we numerically calculated the PT phase modulation for varying pump modulation frequency in the oxygen-filled HC-NCF [20]. The mode fields for the pump and the probe in the HC-NCF are approximated as Gaussian distribution with mode field radiuses of 12.5 μm at 760 nm and 13.5 μm at 1550 nm. The cladding material of the HC-NCF is silica and its central region is filled with 20.8% oxygen balanced in nitrogen. The ambient temperature and gas pressure are assumed to be 293 K and 1 bar, respectively. The thermal parameters of nitrogen are used in solving the thermal conduction equation. The pump is sinusoidally modulated at frequency *f*, which is varied from 2 kHz and 100 kHz. Based on these conditions mentioned above, the amplitude of PT phase modulation as a function of modulation frequency can be obtained. For the convenience of comparison, we selected the amplitude of PT phase modulation at 3 kHz as the reference value and the normalized output is expressed as a level in decibels (dB) by evaluating ten times the common logarithm of the ratio of the simulation results to the reference value, which is represented by the red line in Figure 3. The blue dots are the data obtained from the experiment, which will be described in the next section. At low pump modulation frequencies, the PT phase modulation shows a relatively flat response. At high pump modulation frequencies (e.g., *f* > 15 kHz), due to the slow thermal conduction related to the buffer gas thermal parameters and fiber characteristics, the change of temperature field could not catch up the laser modulation rate, leading to a reduced amplitude of PT phase modulation.

## 3. Experiments and Results

### 3.1. Experimental Setup

**Figure 4:**
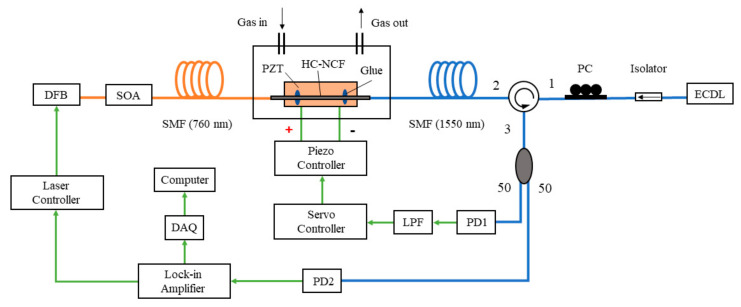
Experimental setup of oxygen detection based on PTI with a HC-NCF. PC, polarization controller; LPF, low pass filter; PD, photodetector; DAQ, data acquisition.

Figure 4 depicts the setup of the PTI-based oxygen detection system using the HC-NCF. We use a distributed feedback (DFB) laser with wavelength around 760 nm and linewidth of ~10 MHz as the pump and a semiconductor optical amplifier (SOA) to amplify pump power to about 30 mW. The pump laser is wavelength-modulated sinusoidally, and at the same time, slowly scanned across the oxygen absorption line at 760.88 nm. The pump beam is delivered into the HC-NCF via the SMF (760 nm), propagates through the HC-NCF, and is eventually blocked by the optical circulator. The probe beam is from an external cavity diode laser (ECDL) with a full-width-half-maximum (FWHM) linewidth of 300 kHz, and its wavelength is fixed to 1550 nm. The probe is delivered to the HC-NCF via the SMF (1550 nm), and the reflected probe from the FPI is detected by PD1 and PD2. The power level of the probe laser is 5 dBm, and the power reaching the photodetectors is –17 dBm. The length of the HC-NCF is 4.3 cm. From Equation (1), the amplitude of PT signal is linearly proportional to the length of the HC-NCF. However, the use of a longer HC-NCF increases the gas filling time. The filling time of the HC-NCF is less than 1 min, as has been demonstrated previously with the same type of HC-NCF but of a slightly longer length [20]. The HC-NCF is mounted on a piezo-electric transducer (PZT) with two fixing points using ultraviolet glue. The cavity length can be servo-controlled via the PZT stretcher, using the DC output component from PD1 as the control signal, to ensure that the FPI is always operating at quadrature at the probe wavelength.

The pump modulation is achieved by modulating the injection current of the DFB laser, which modulates the laser frequency and intensity simultaneously. Since the resultant laser intensity modulation of the pump power has large contribution to the non-absorption background of 1*f* signal, the demodulation of 2*f* signal is preferred in traditional wavelength modulation absorption spectroscopy. However, for the PTI system studied here, only the pump power absorbed by gas molecules will contribute to the PT phase modulation, hence the influence of residual laser intensity modulation on its non-absorption background is negligible. Thus, we demodulate the 1*f* component from PD2 by using a lock-in amplifier, which has the largest amplitude among all the harmonic components of wavelength modulation without strong background signal [21].

### 3.2. Results

**Figure 5:**
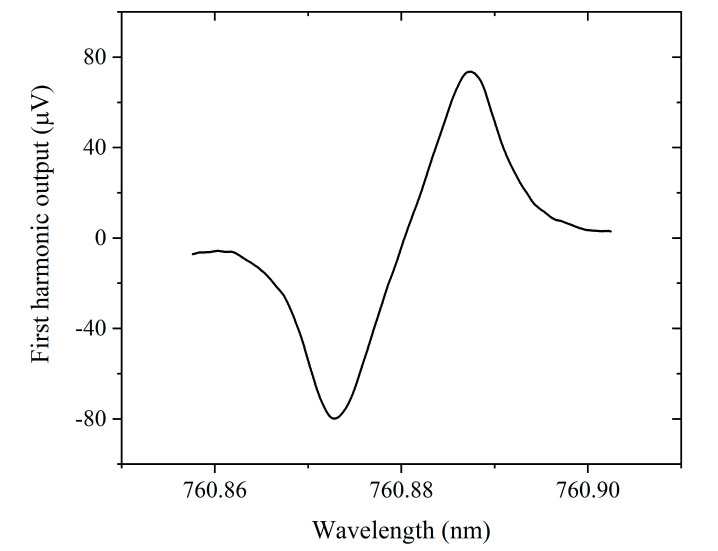
First harmonic output signal for ~20.8% oxygen in air.

All the experiments were conducted under laboratory conditions without temperature/pressure control. The 1*f* signal as a function of modulation frequency from 3 kHz to 50 kHz was measured with laboratory air with an estimated oxygen concentration of ~20.8%. For comparison with numerical simulation, the 1*f* signal is normalized to the value at 3 kHz and expressed in dB, which is represented as the blue dots in Figure 3. Since the PT signal decreases gradually with increasing pump modulation frequency above 15 kHz, we fixed the pump modulation frequency to 15 kHz for subsequent oxygen concentration detection experiments. The first harmonic output is shown in Figure 5. The peak-to-peak amplitude of the 1*f* signal is 153.5 µV when the pump laser is scanned across the absorption line of oxygen. The system noise of 0.25 µV is estimated by tuning the pump wavelength away from the absorption line and being fixed at 760.9 nm. For a lock-in time constant of 1 s with a filter slope of 18 dB/Oct, the signal-to-noise ratio (SNR) is calculated to be ~614, corresponding to the noise equivalent concentration (NEC) of 339 ppm.

**Figure 6:**
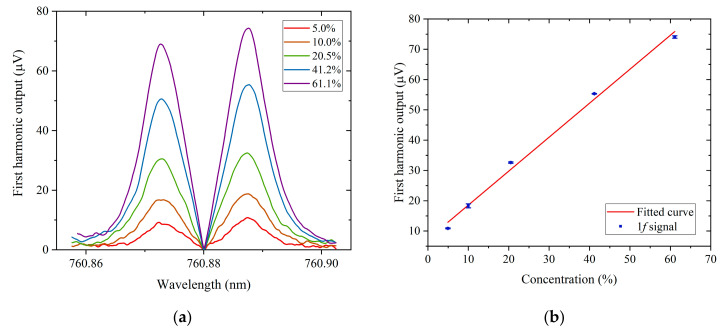
(**a**) First harmonic output signal for 5%, 10%, 20.5%, 41.2% and 61.1% oxygen in nitrogen. (**b**) Peak value of the PT signal as a function of oxygen concentration from 5% to 60%. Error bars show the s.d. of the PT signal.

Figure 6a shows the one set of recorded 1*f* signal for 5%, 10%, 20.5%, 41.2% and 61.1% oxygen in dry nitrogen prepared by mixed gases from commercial gas cylinders. Figure 6b shows the average peak values of the 1*f* signal as a function of oxygen concentration from 5% to 60% balanced in nitrogen. The PT signal increases approximately linearly with oxygen concentration. It needs to be pointed that the PT signal with ~20.8% O_2_ in air is about 2.4 times higher than that in N_2_ and it may be caused by molecular collisions with H_2_O in air, which may improve the relaxation process [18].

**Figure 7:**
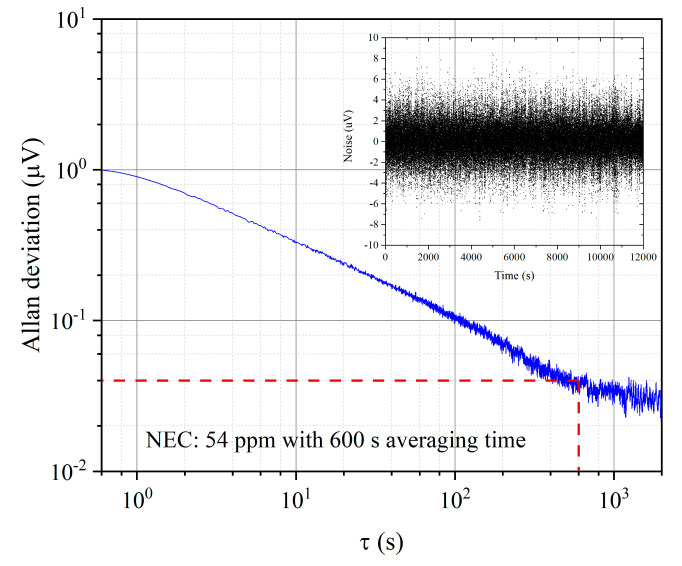
Allan-Werle plot based on the noise data over a period of 3 h, which is shown in the inset.

We also conducted the Allan-Werle deviation analysis to investigate the stability of the detection system. The 1*f* signal for oxygen in atmosphere was recorded for over 3 h when the pump wavelength was away from the absorption line and fixed at 760.9 nm. The time constant of the lock-in amplifier was set to be 100 ms and the sampling rate was 10 Hz. The Allan-Werle plot is shown in Figure 7. The optimal averaging time is ~600 s, at which the noise is ~0.04 µV. The corresponding NEC for a SNR of unity is then estimated to be 54 ppm. The NEC for a 100-s averaging time is 135 ppm.

## 4. Conclusions

In conclusion, we report the first HCF-based oxygen sensor based on PT spectroscopy in a 4.3-cm-long HC-NCF gas cell. The pump laser operating at 760 nm produces PT phase modulation and the probe laser at 1550 nm is utilized to detect the PT phase modulation. The HC-NCF covering both the pump and the probe wavelength bands, and the natural reflections occurring at the joints between the HC-NCF and the transmission fibers form a compact FPI for efficient demodulation of PT phase modulation. By considering the thermal relaxation and thermal conduction of gas in the HC-NCF, the PT phase modulation as a function of pump modulation frequency is investigated theoretically and experimentally. With wavelength modulation and first harmonic detection, getting the detection limit down to 54 ppm with a 600-s averaging time could be achieved, corresponding to NEA of 7.7 × 10^−8^ cm^−1^. The reported LOD of O_2_ is the lowest value achieved in HCF-based O_2_ sensors to the best of our knowledge, and the compact gas cell makes it promising for the in situ monitoring of O_2_.
